# Cerebrospinal fluid protein profiling of inflammatory and neurobiological markers in Lyme neuroborreliosis

**DOI:** 10.1038/s41598-025-06146-y

**Published:** 2025-06-20

**Authors:** Sofie Haglund, Paula Gyllemark, Pia Forsberg, Lars Brudin, Ivar Tjernberg, Anna J. Henningsson

**Affiliations:** 1 Department of Biomedical and Clinical Sciences, Linköping University, Linköping, Sweden; 2 Department of Laboratory Medicine, Region Jönköping County, Jönköping, Sweden; 3 Department of Infectious Diseases, Region Jönköping County, Jönköping, Sweden; 4 Department of Clinical Immunology and Transfusion Medicine, Linköping University, Linköping, Sweden; 5 Department of Clinical Physiology, Region Kalmar County, Kalmar, Sweden; 6 Department of Clinical Chemistry and Transfusion Medicine, Region Kalmar County, Kalmar, Sweden

**Keywords:** Lyme neuroborreliosis, LNB, Proteomics, Protein profiling, Olink, Inflammation, IL10, Clinical microbiology, Infectious-disease diagnostics, Neuroimmunology, Biomarkers

## Abstract

Lyme neuroborreliosis (LNB) is the most common form of disseminated Lyme borreliosis in Europe and North America. There are limitations in existing LNB diagnostics and a lack of reliable objective markers for disease-course. Here, extensive protein profiling with two panels of 184 proteins, was done in the search for new clinically useful diagnostic and prognostic candidate biomarkers. Cerebrospinal fluid (CSF) was collected from patients with definite LNB (*n* = 13) at the time of diagnosis before initiating antibiotic treatment, and at a follow-up one month later. When symptoms were evaluated at a six-month follow-up, six patients had recovered with no persistent symptoms (NPS), and seven experienced delayed recovery with persistent post-treatment symptoms (PS). Orthopedic patients (*n* = 60) served as controls. With the panels used, no protein biomarkers able to differentiate between PS and NPS were identified. However, from a diagnostic perspective, we identified multiple proteins that were differentially expressed between LNB and controls. The majority of them were downregulated following antibiotic treatment, at the one-month follow-up. IL10, TNF, and CCL8 were considered examples of potentially useful candidate biomarkers in both the early diagnostics and in monitoring of treatment response. These markers merit further investigation to understand their utility in relation to other neurological manifestations.

**Supplementary Information:**

The online version contains supplementary material available at 10.1038/s41598-025-06146-y.

## Introduction

Lyme neuroborreliosis (LNB) is caused by infection by bacteria in the *Borrelia burgdorferi* sensu lato complex, hereafter referred to as *Bbsl.* It is the most common form of disseminated Lyme borreliosis in Europe and North America. LNB constitutes around 10–15% of symptomatic *Bbsl* infections^1,2^. It typically presents as subacute meningitis, often with cranial nerve palsy and radiculitis^3^. The diagnostics rely on clinical signs and symptoms, pleocytosis in the cerebrospinal fluid (CSF) and intrathecal production of *Bbsl*-specific antibodies^3^. However, antibodies may take weeks before being measurable in CSF, and thereafter they persist detectable for several years. Therefore, they are less sensitive in the early phase of disease and less specific for ongoing LNB in previously exposed individuals^2,4^. The majority of patients recover after antibiotic treatment, with no persistent symptoms (NPS). However, delayed recovery with long-term post-treatment symptoms (PS) persisting for more than six months, affect, depending on definition, 12–50% of the LNB patients even after adequate antibiotic treatment^5–8^. The etiology and mechanisms behind PS are still poorly understood^9,10^, which calls for deeper investigation.

The *Borrelia* bacteria does not produce any toxins, but studies based on the *B*. *burgdorferi sensu stricto* strain B31, have shown that the hosts inflammatory response to the spirochete is important in the tissue damage process seen in the CNS at LNB^11–14^. Many of these immune-related proteins are detectable in the CSF of patients with LNB^15–23^. The results of previous studies imply that a rapid and strong intrathecal T helper cell (Th) type 1 immune response, followed by Th2- and B cell activation that counter-balances the Th1-response is favorable for recovery^18,19,24^. On the contrary, a high CSF concentration of the pro-inflammatory interleukin IL17A, produced by Th17 cells, has been associated with slower recovery^18,19,25^.

Many of the earlier studies described in the literature are based on a limited number of proteins, selected based on their expected importance in the pathogenesis of LNB, or on smaller protein panels. Although a great number have shown promising results, none of the described candidate biomarkers, except for the B cell-attracting chemokine CXCL13, have so far been widely implemented in the clinical diagnostics of LNB. The utility of CXCL13 as a complement to the traditional laboratory diagnostics is now well documented^26–31^. However, it is present in high concentration in CSF also in other CNS conditions, such as tick-borne encephalitis, herpes simplex encephalitis, multiple sclerosis, neurosyphilis, and autoimmune encephalitis^27,31–34^. In addition, the use of CXCL13 as predictor of disease course in LNB has so far been less successful^18,19,35^. Thus, additional biomarkers are needed to overcome both diagnostic and prognostic limitations, and merits further investigation. A further understanding of the proteins involved in the pathogenesis of LNB is a prerequisite for the identification of supplementary early diagnostic biomarkers, as well as candidate biomarkers associated with PS and in the long run for finding novel therapeutic targets for these patients.

In this explorative study, broad protein profiling was applied to investigate the intrathecal immune response and protein dynamics in LNB patients with different disease courses. We aimed to search for candidate biomarkers or combination of biomarkers to improve laboratory diagnostics and to predict disease course (PS *vs*. NPS) after antibiotic treatment.

## Results

**Fig. 1:**
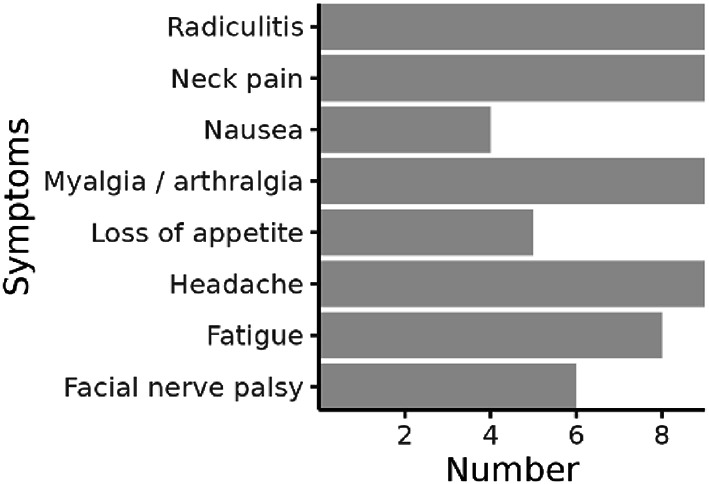
Symptoms in Lyme neuroborreliosis patients (*n* = 13) at the time of inclusion before treatment initiation. Each patient had one or more symptoms.

Thirteen patients with definite LNB were included in this explorative study. Protein profiling was done on CSF samples collected at diagnosis before initiating antibiotic treatment, and at a follow-up visit one month later (Table 1, and Fig. 1). The clinical presentation at the six-month re-examination was used to stratify patients according to PS and NPS. While six patients were judged as having NPS, seven experienced delayed recovery with PS, Table 2. The PS comprised radiculitis (*n* = 2), myalgia/arthralgia (*n* = 3), fatigue (*n* = 4), headache (*n* = 1), and facial nerve palsy (*n* = 1). The baseline characteristics at the two sampling occasions were similar in LNB patients who experienced delayed recovery with PS and those with NPS (Table 2).

As a control group, adult orthopedic patients (*n* = 60) scheduled for either knee or hip joint replacement surgery were recruited. The control group was significantly older than the LNB patients (Table 1), accordingly, the statistical analyses were adjusted for age where appropriate.

**Table 1:** Study population characteristics.

	LNB (*n* = 13)	Controls (*n* = 60)	*p*-value
Female/male	5/8	23/37	0.64
Age, years	52 (39–61)	72 (67–75)	<0.001
CSF mononuclear cells, x10^6^/L	132 (26–306)	1 (0–2)	<0.001
Positive CSF *Bbsl*-specific antibody-index, n (%)	13 (100)	2 (3)	ND
*Bbsl*-specific IgG in serum, n (%)	10 (77)	14 (22)	0.98
CSF/serum-albumin ratio	9.2 (7.8–30.0)	5.7 (4.8–8.3)^a^	<0.001
CSF CXCL13, pg/mL	911 (708–976)	3.0 (0.67–3.6)^b^	<0.001

The table describes median and interquartile ranges. LNB; Lyme neuroborreliosis, CSF; cerebrospinal fluid, *Bbsl; Borrelia burgdorferi* sensu lato complex, ND; not determined, IgG; immunoglobulin G.

^a^The CSF/serum-albumin ratio was available in 46 of the control patients. ^b^The CSF CXCL13 concentration was available in 47 of the control patients.

**Table 2:** Characteristics of patients with Lyme neuroborreliosis stratified according to disease course. Patients with delayed recovery and persistent symptoms (*n* = 7) and those with no persistent symptoms (*n* = 6). The table shows cerebrospinal fluid findings before antibiotic treatment (at diagnosis) and after treatment (one month later).

	PS	NPS	*p*-value
Female/male	3/4	2/4	0.59
Age, years	58 (50–61)	45 (31–59)	0.23
Symptom duration, days	7 (3–14)	14 (8–14)	0.64
**At diagnosis**	(*n* = 7)	(*n* = 6)	
CSF Mononuclear cells, x10^6^/L	132 (26–390)	117 (24–273)	1.00
CSF/Serum-Albumin ratio	12.4 (8.8–26.5)	12.0 (9.2–38.12)	1.00
CSF CXCL13, pg/mL	911 (835–931)	931 (252–992)	0.95
**One month**	(*n* = 7)	(*n* = 5)^a^	
CSF Mononuclear cells, x10^6^/L	14 (10–28)	8 (2–31)	0.34
CSF/Serum-Albumin ratio	6.5 (5.4–10.1)	6.1 (5.0–12.2)	0.88
CSF CXCL13, pg/mL	86 (26–110)	28 (2.1–79)	0.27

The table describes median and interquartile ranges. PS: persistent symptoms; NPS: no persistent symptoms; CSF; cerebrospinal fluid. ^a^Data missing for one patient rejecting sampling at the one month follow-up.

Protein profiling of CSF samples was done by the Olink^®^ Inflammation and the Olink^®^ Neurology panels. After pre-processing of raw-data, 155 of the 184 analysed proteins remained for further analysis of differentially expressed proteins (DEP), Supplementary Table S1 and S2.

### Differentially expressed proteins in LNB patients at diagnosis compared with controls

**Fig. 2:**
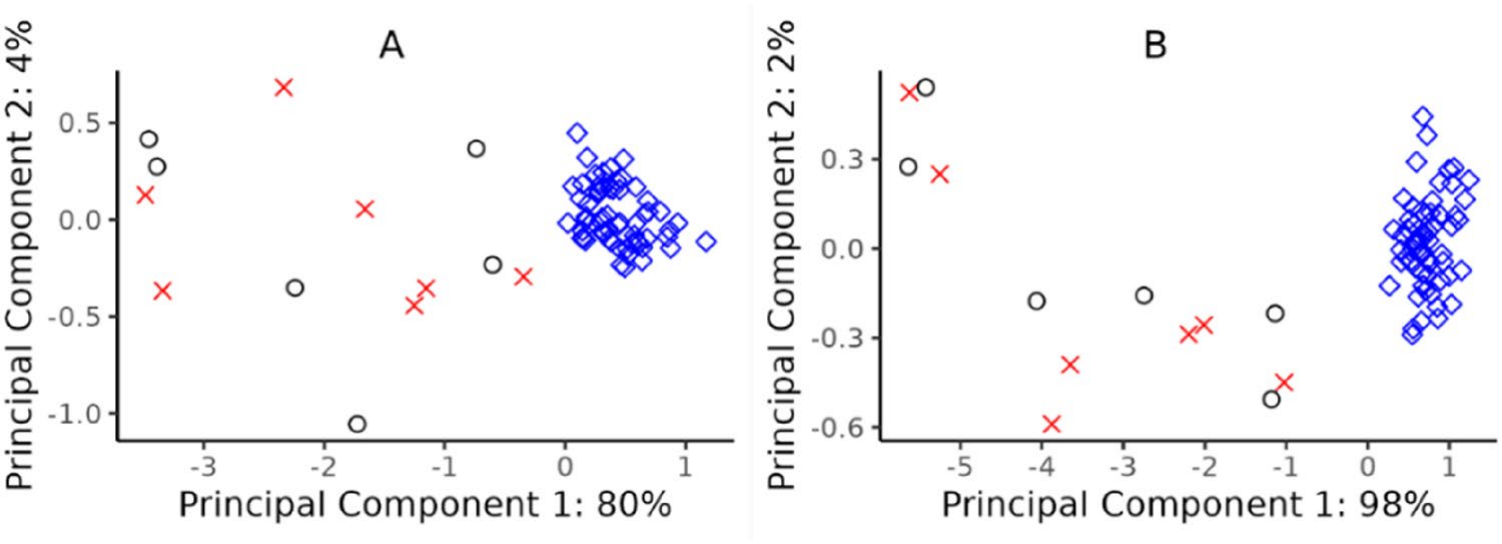
Principal component analysis (PCA) of the normalized protein expression values (log2 scale) of the 46 most upregulated proteins in patients with Lyme neuroborreliosis at diagnosis (*n* = 13) *vs*. controls (*n* = 60). (**A**) PCA based on 46 differentially expressed proteins. (**B**) PCA based on IL10 and TNF only. Red crosses; patients with persistent symptoms, black circles; patients with non-persisting symptoms, blue squares; controls.

At the time of diagnosis, before treatment initiation, 77 DEP were observed in LNB patients (*n* = 13) when compared with controls (*n* = 60), 76 proteins were upregulated, 46 of them with a fold-change (FC) > 2 (Supplementary Table S3). A principal component analysis of the 46 proteins showed that the proteins with strongest association (among all significant proteins) with the separation along the first principal component were IL10 (*p* = 4.19 × 10^−50^) and TNF (*p* = 1.26 × 10^−49^). Using these two proteins only, 98% of the variation in data along the first principal component was explained (Fig. 2). The diagnostic performance was evaluated by ROC-curve (not illustrated). In this study cohort the separate areas under the curve for IL10, TNF and CXCL13 were all 1.00.

**Fig. 3:**
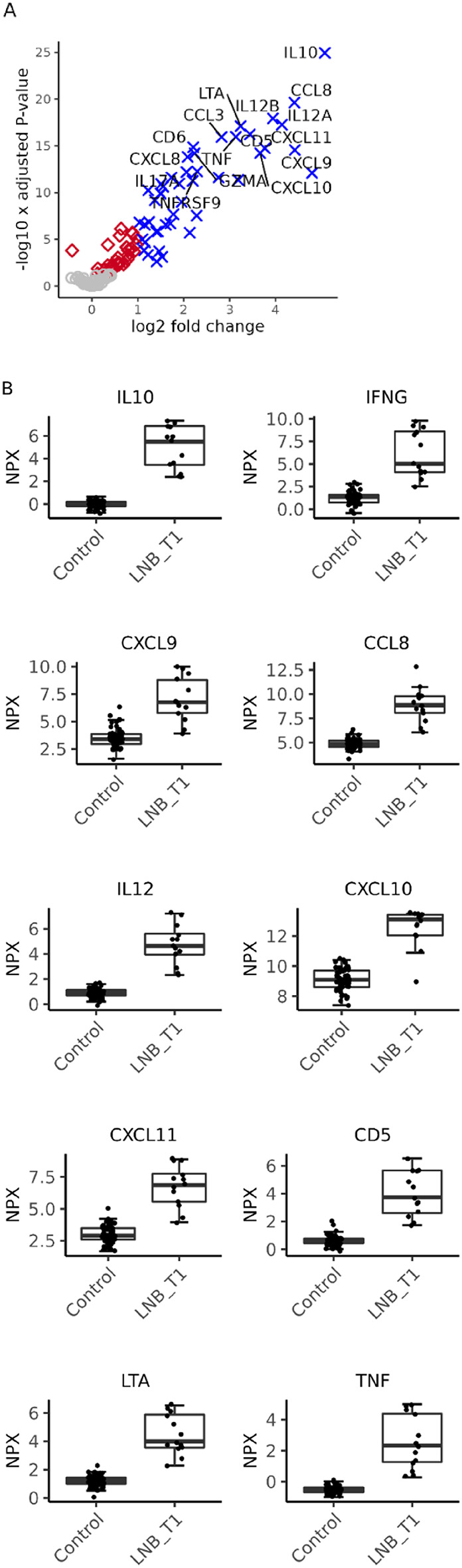
Differentially expressed proteins at the time of diagnosis in Lyme neuroborreliosis (LNB) vs. controls. (**A**) Volcano-plot illustrating the differentially expressed proteins in LNB patients (*n* = 13) *vs*. controls (*n* = 60). Blue; proteins upregulated at least 2-fold with a false discovery rate adjusted p-value (FDR) < 0.05, red; proteins with a FDR < 0.05, gray; non-significant proteins. (**B**) Normalised protein expression values (NPX, log2 scale) of the ten proteins with highest fold change in LNB *vs*. controls (log FC > 3, *p* < 8.09 × 10^−13^). Proteins are described by their gene symbol. LNB_T1; Lyme neuroborreliosis patients at diagnosis, before treatment. IL12 was represented by IL12B.

The proteins with the highest average FC among the most significantly upregulated proteins, and thus strongest diagnostic candidate makers were IL10, IFNG, CXCL9, CCL8, IL12, CXCL10, CXCL11, CD5, LTA, IL6, and TNF (log FC > 3, *p* < 8.09 × 10^−13^), exemplified in Fig. 3.

**Fig. 4:**
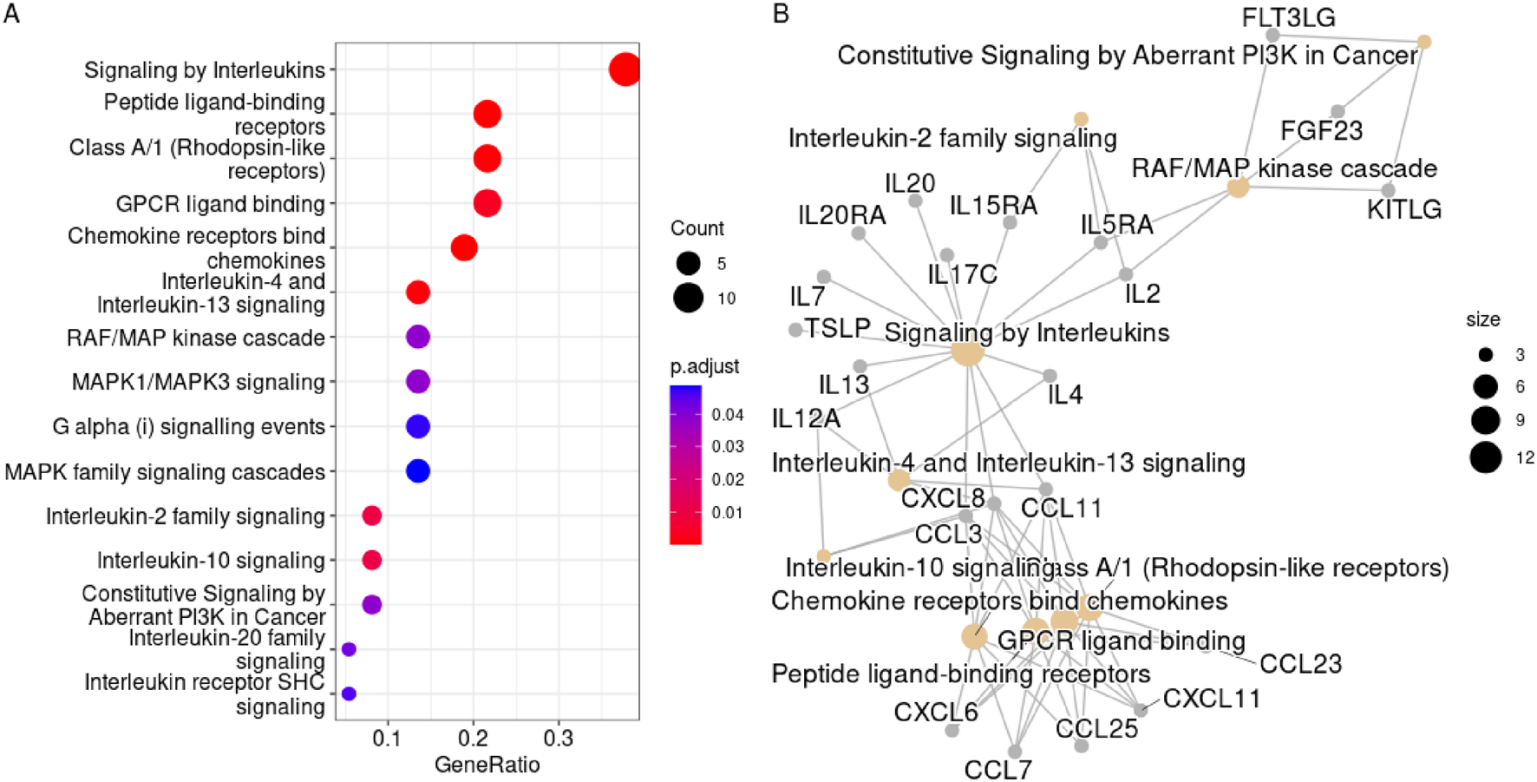
Over-representation analysis. Enriched Reactome terms in the 46 differentially expressed proteins with a fold-change *≥* 2 in the comparison between Lyme neuroborreliosis patients at diagnosis, before treatment initiation (*n* = 13), and controls (*n* = 60). (**A**) Dot plot of enriched terms in the 46 proteins, and (**B**), cnet plot over the, for clarity, top ten enriched terms. Proteins are described by their gene symbol.

The ORA analysis of the 46 most DEP between LNB and controls suggested an accumulation of proteins of pathways of signal transduction via peptide ligand binding receptors (GPCR and rhodopsin- like receptors) and in intracellular signaling pathways downstream of these receptors involving mitogen activated protein (MAP) kinases, and phosphoinositide in addition to interleukin signaling pathways (Fig. 4, Supplementary Table S4).

While multiple DEP were identified between LNB patients and controls, none of the proteins included in the protein panels used here were able to prospectively differentiate between the two groups of LNB patients showing PS *vs.* NPS at 6 months (Supplementary Table S5). At 12 months, radiculitis, myalgia and/or arthralgia and facial nerve palsy remained in four patients. Although a small group, we observed higher levels of IL17A, CD5, TNFSF10 and TNFS14 at diagnosis in these patients compared with those with NPS. When investigating the protein profiles in the one-month samples, these differences were no longer detectable (Supplementary Table S6).

### Protein dynamics following antibiotic treatment within the Lyme neuroborreliosis cohort

**Fig. 5:**
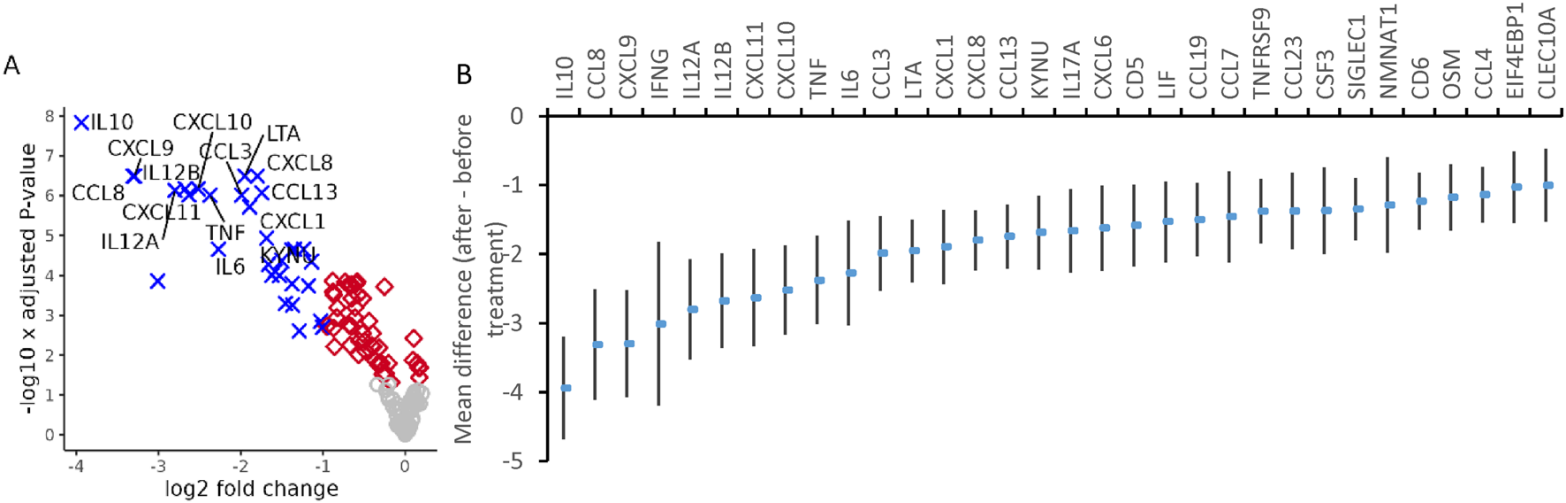
Protein dynamics between diagnosis and the one month follow-up. (**A**) Volcano-plot illustrating the paired comparison of protein expression levels in Lyme neuroborreliosis patients (*n* = 12) at the one-month follow-up (after antibiotic treatment) *vs.* the time of diagnosis. One patient rejected sampling at the one-month follow-up. Blue; proteins downregulated at least 2-fold at follow-up, with a false discovery rate adjusted *p*-value (FDR) < 0.05, red; proteins with a FDR < 0.05, gray; non-significant proteins. (**B**) The mean difference between protein expression levels at the one-month follow-up and at the time of diagnosis with 95% confidence intervals, illustrated for the 32 proteins with a foldchange < − 2. Proteins are described by their gene symbol and sorted based on significance.

**Fig. 6:**
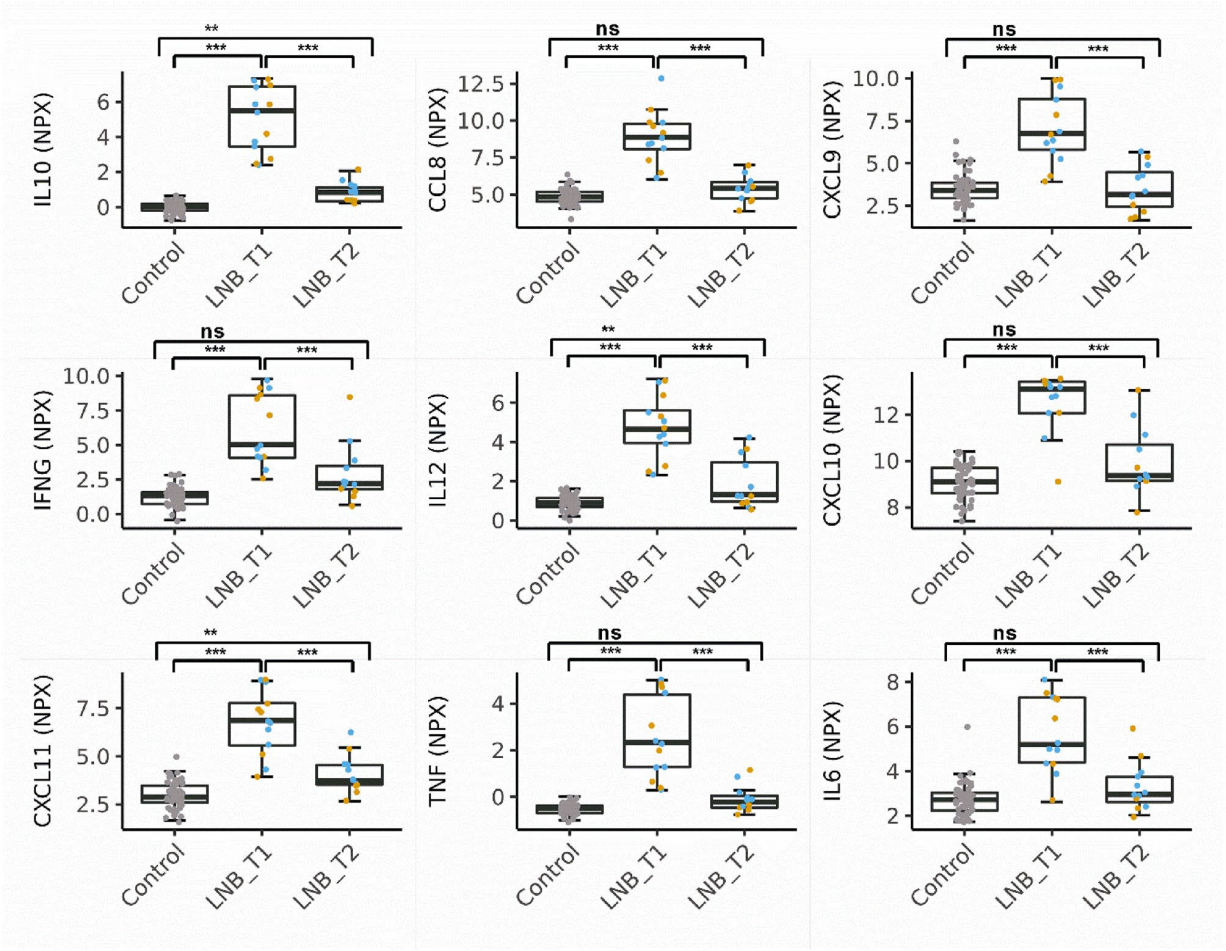
Protein levels in controls, patients with Lyme neuroborreliosis (LNB) at diagnosis and at the one-month follow-up. The normalized protein expression values (NPX, log2 scale) of the nine most differentially expressed proteins (negative foldchange < − 2, *p* < 2.24 × 10^−5^) at the one-month follow-up (after treatment) in LNB patients *vs*. the paired samples taken before treatment (*n* = 12). Protein levels of the control cohort, included for comparison (*n* = 60). Proteins are described by their gene symbol. LNB_T1 (*n* = 13); LNB patients before antibiotic treatment, LNB_T2 (*n* = 12) LNB patients at the one month follow-up. IL12 is represented by IL12B. Blue; persistent symptoms, orange; non-persistent symptoms, ns; *p* > 0.05, **p < 0.01, ***p < 0.001.

To identify proteins associated with the response to antibiotic treatment, the overall protein dynamics was assessed by comparing the paired samples taken before and after treatment, at the one-month follow-up, in the LNB cohort. Eighty-one proteins were downregulated following treatment, 32 with a negative FC < − 2 compared with the protein expression levels before treatment initiation (Fig. 5, further detailed in Fig. 6, and Supplementary Table S7). All 32 of these most downregulated proteins were among the 46 most upregulated proteins with a FC > 2, compared with controls, at the time of diagnosis. Thus, the 32 most DEP following treatment represented the same biological pathways upregulated in the initial comparison as illustrated by the ORA), Fig. 4.

### Protein dynamics following treatment in Lyme neuroborreliosis patients with persistent symptoms and in those with non-persistent symptoms

The possibility to use the protein dynamics within respective group of patients with PS and NPS, as a tool to predict disease course and to identify patients in need of extra monitoring was explored. However, statistically there was no difference in the protein dynamics between the groups of patients with different disease courses (PS *vs*. NPS) with the group sizes and protein panels used here (Supplementary Table S8).

### CSF mononuclear cell concentration and protein expression levels

The CSF mononuclear cell concentration correlated positively with 32 proteins at the time of diagnosis. At the one-month follow-up, after antibiotic treatment, CD6, TNFRSF14, CD5, IFNG, TFNRSF9, LTA, and CRTAM correlated positively with the CSF mononuclear cell concentration measured at this time point (Supplementary Table S9). No correlation between protein levels and the symptom duration before diagnosis was observed (Supplementary Table S10).

## Discussion

The CNS immune response to the *Borrelia* spirochete is orchestrated by Th1, Th2, and Th17 lymphocytes as well as of CNS- derived cells such as microglia, astrocytes and oligodendrocytes. The diagnostics of LNB is challenging and there is a need for additional supplementary biomarkers, and biomarkers characteristic for the varying disease course. Here we used two protein panels comprising 184 proteins, associated with inflammation and neurobiology, to get a further insight in the immune response and protein dynamics in LNB from a diagnostic perspective, but also in order to explore the feasibility of protein profiling in the search for candidate markers associated with disease course following treatment.

We identified 46 candidate protein biomarkers in CSF able to differentiate between LNB patients, before treatment initiation, and controls. Following treatment, the expression level of the majority of these proteins (*n* = 32) were downregulated at least 2-fold at the one-month follow-up, when compared with the level at diagnosis, which holds promise for future studies. So far, there are no reliable independent diagnostic tools to identify or predict the risk of a delayed recovery with PS in LNB. These symptoms are often subjective, and without objective evidence for a persistent *Bbsl* infection, but have a negative impact on the quality of life of affected patients^9,10,36^. We were not able to identify any biomarkers that could aid in the identification of these patients, when PS was defined based on the clinical presentation at the six-month follow-up, neither when protein levels were explored at diagnosis nor when investigating the protein dynamics over time. IL17A, which was associated with a delayed recovery in our previous report^19^, did not pass correction for multiplicity here (Supplementary Table S5). Results similar to ours have been reported in studies where CSF was collected in the clinical phase with PS^37^. However, higher levels of IL17A (FC > 2), CD5 (FC > 2), TNFSF10 and TNFS14 were observed at diagnosis in the group of patients who experienced PS at 12 months compared with those with NPS. These differences were no longer detectable in the one-month samples following treatment. The smaller number of patients presenting with PS at 12 months (*n* = 4) limits the possibility to draw conclusions from this observation. CD5 is, in addition to being involved in regulating T cell and B cell survival, also expressed on a subset of IL10 producing B cells (B10) with a negative regulatory function. They are involved in the supression of Th1 and Th17 responses. The function of soluble CD5 is however not yet fully known, as reviewed in^38^. The TNFSF molecules are involved in diverese processes that control cell proliferation, survival and inflammation. TNFSF10 (TRAIL) is generally considered pro-apoptotic and show neurotoxic properties in many neurological inflammatory conditions. However, it also is involved in the clearance of infected antigen presenting cells by interaction with their death receptors, reviewed in^39,40^. TNFSF14 (LIGHT) is a CD28 independent costimulatory molecule expressed on activated T cells reciding in CNS and has also an important role in controlling activated microglia^40^.

In the search for new early diagnostic biomarkers for LNB we aimed at an absolute FC > 2 when evaluating the DEP between patients and controls. The majority of the candidate biomarkers identified stemmed from the Inflammation panel. Granzyme A (GZMA) was the only DEP from the Neurology protein panel among the top 20 DEPs with a FC > 2. However, it is a cytotoxic effector-protein involved in the immune response^41^. The most upregulated proteins in LNB, with a log FC > 3 *vs*. controls were; IL10, IFNG, CXCL9, CCL8, IL12, CXCL11, CXCL10, CD5, LTA, IL6, and TNF. Following age, the candidate biomarkers IL10 (anti-inflammatory) and TNF (pro-inflammatory), showed the strongest correlation with phenotype based on the unsupervised PCA.

IL10 is, due to its inhibitory effect on T cells and macrophages central to the resolution of the first Th1 mediated pro-inflammatory response in LNB^42–44^. The identification of IL10 as a potential diagnostic biomarker corroborates our previous findings based on smaller protein panels^23,45^ as well as studies by others^16,46,47^. IL10 is produced by many cells types. In CNS glial cells it is induced by TNF and IL6^43^, and in regulatory B cells its production is modulated by the inflammatory milieu and stimulation of antigen receptors^48^. The concentrations of TNF, IL6 and CXCL8, are all increased in the early response to *Bbsl* as demonstrated both *in vitro* and in rhesus macaque monkey models of LNB exposed to *B. burgdorferi senso strictu* strain B31^11–14^. These cytokines play important role in the regulation of neuronal and glial cell apoptosis^11,13,44,49^. The high concentrations observed *in vivo* at diagnosis in our LNB cohort are in agreement with many other reports^16,23^. The concentration of both IL6 and CXCL8 have been reported to be similar in LNB and in viral meningitis^46^ and from a pathophysiological perspective, IL6 has been described as a nonspecific biomarker of meningitis^50^.

Overall, the biomarkers identified here corresponded with an innate and Th1- driven immune-response, characterized by the upregulation of CCL8, CCL3, IL12 (IL12B p40, IL12A p35), TNF, LTA, IFNG, CXCL10, CXCL11, as well as a Th17 response represented by IL17A, in addition to the counterbalancing IL10, at diagnosis^16,18–20,24,25,37,51–53^.

The ORA confirmed the protein-by-protein analysis by the enrichment of proteins of pathways involved in peptide ligand-binding G protein coupled receptors within the Class A/1 rhodopsine receptors. The large family of G-protein coupled receptors are closely linked to the phosphoinositide, Src and mitogen-activated protein (MAP) kinase pathways regulating the synthesis of inflammatory cytokines, cellular differentiation, apoptosis and proliferation^54,55^.

By the pairwise analysis of post-treatment *vs*. pre-treatment samples it was shown that the majority of DEP at diagnosis (when compared with controls) were downregulated following treatment with antibiotics. Therefore, we considered for example IL10, TNF, and CCL8 as potential candidate biomarkers of both disease and response to treatment, in addition to CXCL13 reported earlier for the same patient cohort^19^. These proteins were also among the most DEP by treatment in the report by Pietikäinen et al. when using another protein panel^16^. Even though these proteins were associated with both disease and response to antibiotics they represent proteins generally involved in the inflammatory response to bacterial infections. However, as biomarkers they merit further investigation in combination with other clinical parameters used in the LNB diagnostics. CXCL9, CXCL10 and CXCL11 were highly upregulated in LNB *vs*. controls at diagnosis and decreased following antibiotic treatment. However, their utility as biomarkers in the context of LNB may be questioned, as concentrations similar to those seen in LNB have also been documented in the CSF of tick-borne encephalitis patients^16,56^.

Our study comprised a well characterized cohort of definite LNB patients. They were followed prospectively and clinical symptoms were evaluated systematically at diagnosis, and after 1, 6 and 12 months. The control cohort (all above 52 years of age) had a similar seroprevalence (23%) as found in other endemic areas of Sweden; around 20–25%^57^, and thus represents the real life situation. Two controls had positive *Bbsl* antibody-index but did not have any clinical or biochemical signs of ongoing active borreliosis or other neurological conditions. They were therefore included in the control cohort.The study is limited by the small number of patients and must be regarded as a pilot exploring the utility of largescale targeted protein profiling in the search for candidate biomarkers aiding in the diagnosis and monitoring of treatment efficiency in LNB. Our results need further confirmation in larger populations, also including other infectious and non-infectious neurological conditions, to further support the specificity of the candidate biomarkers identified. The samples had been stored at −80℃ for > 10 years, which may possibly affect the measured protein concentrations. However, all samples were treated the same way. Thus, the detected differences between study groups should still be valid. We were not able to explain differences in disease course by the use of the selected protein panels. Although they comprise a fairly large number of proteins, they are designed to identify neurological and inflammatory proteins only. They don’t allow an unsupervised proteomics approach. In this regard, it is therefore possible that other proteins or pathways, not detected by the protein panels, could differentiate between patients with PS and NPS.

The reporting of PS in patients with delayed recovery is to a large degree subjective and based on patients’ experiences, which of course may affect the results. At the 12-months follow-up the majority of patients (70%) had recovered completely. Although limited, our protein data suggest that there is a normal variability in the time to recovery, not explained by the studied proteins, and that the majority of patients are healed within a year. However, it is also important to consider yet unidentified biomarkers as well as other underlying clinical conditions in cases with long-term complaints.

It is generally difficult to compare results between largescale protein profiling studies. Concordance is affected by panel content, the control cohort selected, the preprocessing and filtering of data, the statistical methods used etc. However, even though different statistical approaches were used here and in other previous quite large protein profiling studies^16,23,47^, we all identified similar top candidate biomarkers with potential to aid in the diagnostics and monitoring of LNB.

## Conclusion

By the use of two broad protein panels of inflammatory and neurological markers, we identified multiple DEP in the comparison between LNB patients and controls. The majority was downregulated at the one month follow-up, after treatment. These candidate markers may aid in the diagnostics of LNB, and merit further investigations in larger cohorts of patients including other neuro-infections as well as other neurological conditions. However, we were not able to identify any predictive CSF biomarkers allowing the differentiation between patients with PS and NPS at six months already at the time of diagnosis or at the one-month follow-up. This highlights the difficulty in establishing stringent biomarkers to identify these patients. Our results confirm the interplay between Th1, Th2, and Th17 cells in LNB and add new knowledge to the immune-regulation. Further in-depth studies will reveal if the proteins identified here may serve as clinically useful biomarkers in the diagnosis and monitoring of LNB.

## Materials and methods

### Patients

CSF samples were collected from patients aged *≥* 18 years, admitted under the suspicion of LNB to the Department of Infectious Diseases, Region Jönköping County, between 2005 and 2010. Only patients with definite LNB, defined according to the European Federation of the Neurological Societies criteria, were included;

(i) neurological symptoms compatible with LNB.
(ii) pleocytosis in CSF (mononuclear cells > 5/µL CSF).
(iii) intrathecally produced anti-*Borrelia* specific antibodies (elevated *Bbsl* antibody index)^3^.

Samples for protein profiling were collected in the acute phase as part of the routine diagnostic procedure and at a one-month follow-up visit. In between, all patients were treated with oral doxycycline 200–400 mg/day for 10–14 days according to national guidelines. All CSF samples were immediately stored at − 80℃ without centrifugation upon arrival at the laboratory. Patients’ symptoms were systematically assessed by a specialist in infectious diseases at inclusion, and after 1, 6, and 12 months. In total 13 LNB patients were included (Table 1, and Fig. 1). The six-month evaluation was used to categorize them as having either NPS (*n* = 6) or delayed recovery with PS (*n* = 7), Table 2. PS was defined as symptoms at inclusion remaining fully or partially at the six-month follow-up^3,19,36^, but was also evaluated at the 12-month visit.

The control group comprised adult orthopedic patients (*n* = 60) recruited from the Department of Orthopedic surgery, Region Jönköping County, between 2016 and 2018. These patients were scheduled for either knee or hip joint replacement surgery and CSF was retrieved during spinal anesthesia. All CSF samples were immediately stored at − 80℃ without centrifugation upon arrival at the laboratory. Exclusion criteria were; autoimmune disease, immunosuppressive treatment, neurological disease, diabetes and malignancy.

### CSF protein profiling

CSF samples were analysed by proximity extension assays provided by the Clinical Biomarkers facility, Science for Life Laboratory, Uppsala University, Sweden, according to the manufacturers instructions. In the study, the Olink^®^ Inflammation panel v.3021 and the Olink^®^ Neurology panel v.8011 were used based on the hypothesis that LNB patients show different biomarker signatures compared with control patients, and that the immune regulation in CNS differs between LNB patients with different disease courses. The protein panels covered 184 proteins in total. Protein levels were expressed on a relative log2 scale with arbitrary units presented as normalized protein eXpression (NPX). A protein was included in the statistical analysis if it was expressed in at least 50% of samples within at least one of the two groups studied. The reported NPX value was used without any adjustment for samples where the reading was below the limit of detection of the included proteins. The proteins included are described by the protein (gene) symbol. The corresponding full protein names are described with their unique protein identification number, www.uniprot.org^58^, in Supplementary Table S1.

### Serum and CSF routine analyses

Patient and control samples were analysed as previously described^19^. Briefly, the CSF mononuclear cell count, the CSF-albumin/serum-albumin ratio as measure of the blood brain barrier integrity, and the immunoglobulin G (IgG) index were determined. The IDEA Lyme Neuroborreliosis kit (Dako Cytomation) was used for paired serum and CSF samples to determine the intrathecal production of *Bbsl*-specific antibodies and the *Bbsl*-specific CSF/serum antibody index.

### Statistics

For the comparison of demographic data between groups the Mann-Whitney’s U-test and Fisher’s exact test for categorical data were used. In all comparisons two-sided tests were used and considered statistically significant if *p* < 0.05. Median and interquartile (IQR) values are described for patient characteristics. These statistical analyses were performed in SPSS Statistics v.27 (IBM Corp, Armonk, NY). The analysis for DEP was performed in R v.4.2.0 in RStudio Pro Build 353.pro20^59^ using linear modelling and the limma package^60^. This strategy has been shown powerful in detecting differences in protein expression levels^61,62^. An empirical Bayes moderated t-test adjusted for age, was used to assess significant differences from zero for each individual contrast. Thus, no FC cut-off was used when establishing significant results. The *p* values were corrected for false discovery rate according to Benjamini and Hochberg^63^, and proteins with corrected *p* values < 0.05 were considered to be significantly DEPs. Following statistical analysis, proteins with an absolute FC > 2 were considered as potential candidate biomarkers. For paired comparisons the duplicateCorrelation function in limma was applied. ORA was done by the R package ReactomePA^64^ and the Reactome database v79 as of April 2022 to explore the biological context of DEP. Enriched terms were visualized by dot plot and proteins involved in the most significant terms by cnet plot via the enrichplot package. Graphs and volcanoplots were prepared using the R package ggplot2 v.3.3.6.

## Electronic supplementary material

Below is the link to the electronic supplementary material.

Supplementary Material 1

## Data Availability

The datasets used and/or analysed during the current study are available from the corresponding author on reasonable request.

## Abbreviations

*Bbsl*: Borrelia burgdorferi sensu lato (s.l.) complex
CNS: Central nervous system
CSF: Cerebrospinal fluid
DEP: Differentially expressed proteins
FC: Fold change
LNB: Lyme neuroborreliosis
NA: Not applicable
NPS: No persistent symptoms
ORA: Overrepresentation analysis
PCA: Principal component analysis
PS: Persistent symptoms
Th: T-helper cell
